# Unravelling the Effects of the Mutation m.3571insC/*MT-ND1* on Respiratory Complexes Structural Organization

**DOI:** 10.3390/ijms19030764

**Published:** 2018-03-07

**Authors:** Luisa Iommarini, Anna Ghelli, Concetta Valentina Tropeano, Ivana Kurelac, Giulia Leone, Sara Vidoni, Anne Lombes, Massimo Zeviani, Giuseppe Gasparre, Anna Maria Porcelli

**Affiliations:** 1 Dipartimento di Farmacia e Biotecnologie (FABIT), Università di Bologna, Via Francesco Selmi 3, 40126 Bologna, Italy; annamaria.ghelli@unibo.it (A.G.); concettavalentina.tropeano@gmail.com (C.V.T.); giulia.leone5@studio.unibo.it (G.L.); annamaria.porcelli@unibo.it (A.M.P.); 2 Dipartimento Scienze Mediche e Chirurgiche (DIMEC), U.O. Genetica Medica, Pol. Universitario S. Orsola-Malpighi, Università di Bologna, via Massarenti 9, 40138 Bologna, Italy; ivana.kurelac@unibo.it (I.K.); giuseppe.gasparre@gmail.com (G.G.); 3 Medical Research Council, Mitochondrial Biology Unit, Cambridge CB2 0XY, UK; Sara_Vidoni@dfci.harvard.edu (S.V.); mdz21@mrc-mbu.cam.ac.uk (M.Z.); 4 Inserm U1016, Institut Cochin, F-75014 Paris, France; anne.lombes@inserm.fr; 5 Centro Interdipartimentale di Ricerca Industriale Scienze della Vita e Tecnologie per la Salute, Università di Bologna, 40100 Bologna, Italy

**Keywords:** respiratory complex I, *MT-ND1*, ND1, OXPHOS, respiratory complexes, supercomplexes, mtDNA mutation, respirasome, mitochondria

## Abstract

Mammalian respiratory complex I (CI) biogenesis requires both nuclear and mitochondria-encoded proteins and is mostly organized in respiratory supercomplexes. Among the CI proteins encoded by the mitochondrial DNA, NADH-ubiquinone oxidoreductase chain 1 (ND1) is a core subunit, evolutionary conserved from bacteria to mammals. Recently, ND1 has been recognized as a pivotal subunit in maintaining the structural and functional interaction among the hydrophilic and hydrophobic CI arms. A critical role of human ND1 both in CI biogenesis and in the dynamic organization of supercomplexes has been depicted, although the proof of concept is still missing and the critical amount of ND1 protein necessary for a proper assembly of both CI and supercomplexes is not defined. By exploiting a unique model in which human ND1 is allotopically re-expressed in cells lacking the endogenous protein, we demonstrated that the lack of this protein induces a stall in the multi-step process of CI biogenesis, as well as the alteration of supramolecular organization of respiratory complexes. We also defined a mutation threshold for the m.3571insC truncative mutation in mitochondrially encoded NADH:ubiquinone oxidoreductase core subunit 1 (*MT-ND1*), below which CI and its supramolecular organization is recovered, strengthening the notion that a certain amount of human ND1 is required for CI and supercomplexes biogenesis.

## 1. Introduction

Mitochondrial complex I (CI) (reduced nicotinamide adenine dinucleotide NADH: ubiquinone oxidoreductase, EC.1.6.5.3) is the first energy-transducing multiprotein component of the oxidative phosphorylation (OXPHOS) system. Its main function is to couple proton translocation with electron transfer from NADH to ubiquinone, thus contributing to generate the mitochondrial membrane electrochemical proton gradient, the driving force for ATP synthesis by complex V (CV) [1]. Mammalian CI is composed of 44 subunits, seven of which (ND1–6 and ND4L) are encoded by mitochondrial DNA (mtDNA) and constitute the hydrophobic membrane arm. The remaining 37 subunits are coded by the nuclear genome (nDNA) and assembled within both the hydrophilic and hydrophobic arms, to form a slightly opened L-shape structure [2]. Three functional and structural distinct modules can be identified: the N-module that provides NADH oxidation, the Q-module that allows the reduction of ubiquinone, and the P-module for proton pumping. Complex I biogenesis results from a dynamic assembly process during which both nDNA- and mtDNA-encoded proteins are organized to form subcomplex intermediates, which are subsequently combined into the functional holoenzyme [3]. Moreover, at least 13 specific molecular chaperones are required for the proper assembly process, increasing the complexity of such mechanism [4]. Among CI mtDNA-encoded subunits, ND1 is a core protein evolutionary conserved from bacteria to mammals [5]. Recent structural analysis of mammalian CI revealed that, similar to bacteria and yeast, ND1 is placed at the interface between the two arms, where it is involved in the formation of the fourth proton pumping site. Together with ND4L and ND6, it is part of the ubiquinone binding site and is likely to couple the electron transfer with the conformational changes in the membrane domain needed to drive proton translocation across the membrane [5,6,7,8]. These data strongly support the idea that ND1 has a crucial role in maintaining the structural and functional interaction among CI modules.

It is now well established that a large portion of the mitochondrial respiratory chain complexes is organized in supramolecular assemblies, or supercomplexes. Approximately 90% of mammalian CI is found associated with respiratory complex III and IV (CIII and CIV) with different stoichiometry forming the supercomplex CI+CIII_2_, or the respirasome (CI+CIII_2_+CIV_n_) [9,10,11,12]. More recently, it has been also described a megacomplex composed of dimers of CI, CIII and CIV (CI_2_+CIII_2_+CIV_2_) [13]. The existence of such supramolecular structures is now clearly established, although their functional role is still uncertain [14]. Respiratory CI dysfunctions may arise from either mtDNA and nDNA mutations, and they have been involved in mitochondrial disorders, neurodegenerative diseases, aging, and tumor progression [15]. One of the peculiar features of mitochondrial genetics is the existence of the threshold effect. Since mtDNA is present in multiple copies within a cell, mutant and wild type molecules can co-exist in heteroplasmy, whereas the condition in which all mtDNA molecules are identical (either mutant or wild type) is termed homoplasmy [16]. As a consequence, a heteroplasmic mutation must surpass a certain mutation threshold (typically 70–80%) to uncover a biochemical and/or clinical phenotype [17], and thus most mtDNA mutations are considered recessive.

In this work, we investigated the effects of the m.3571insC/*MT-ND1* frameshift mutation at different mutation loads on the molecular organization of respiratory CI and supercomplexes. We here confirmed that the lack of ND1 induced a complete disassembly of human CI and CI-containing supercomplexes and, for the first time, that low amounts of wild type ND1 are sufficient to recuperate this defective phenotype. These findings allowed us to set the genetic threshold for such recovery in the very tight range between 93% and 85% of mutant mtDNA. Further, we demonstrated that when the amount of fully assembled CI is limiting, respirasome is preferentially assembled among the supramolecular structures in which respiratory complexes can aggregate.

## 2. Results

### 2.1. Ablation of ND1 Induced by Homoplasmic m.3571insC/MT-ND1 Mutation Determines a Stall in Complex I Assembly

**Figure 1:**
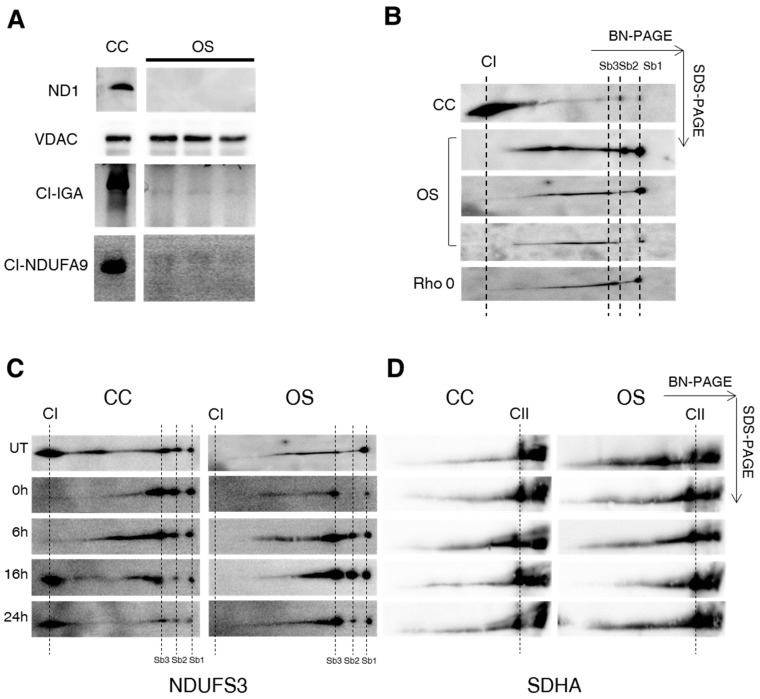

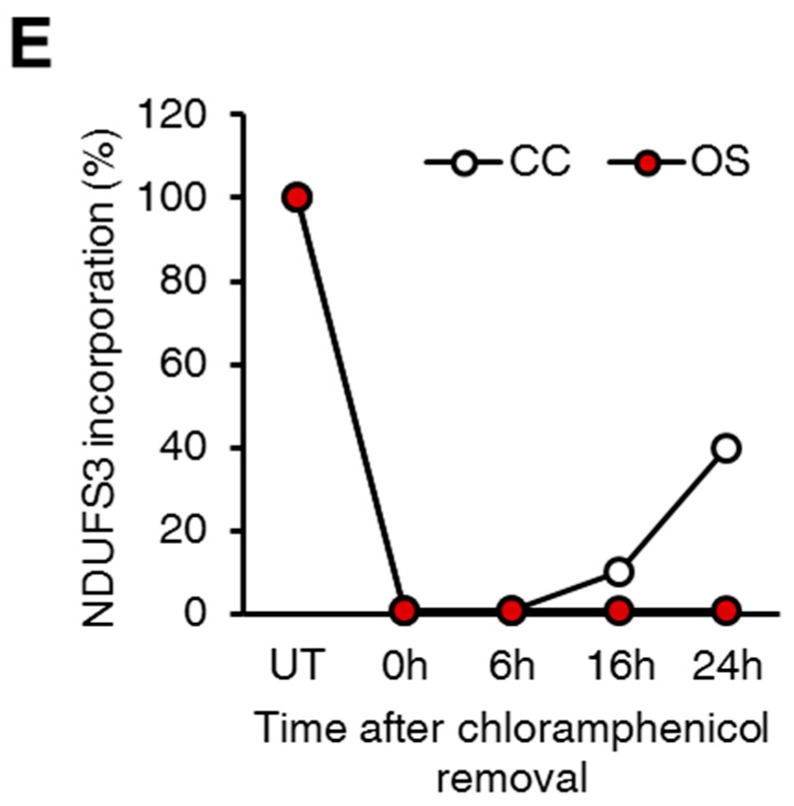
Human ND1 is necessary for CI assembly and stability. (**A**) Immunoblotting of ND1 levels in crude mitochondria obtained from control (CC) and three different ND1-null cybrids (OS). One representative experiment of three is shown. Complex I In-Gel Activity (CI-IGA) and western blot analysis of isolated CI using an antibody against the NDUFA9 subunit performed on isolated respiratory complexes separated by hrCNE. VDAC1 was used as a loading control. (**B**) 2D BN/SDS-PAGE performed on mitochondria-enriched fractions obtained from control (CC), three different ND1-null (OS) and mtDNA depleted (Rho 0) cybrids followed by western blot analysis using an antibody against NDUFS3. Fully assembled CI and sub-complexes (Sb1-2-3) are indicated by dotted lines. (**C**) 2D BN/SDS-PAGE followed by western blotting using an antibody against NDUFS3. Mitochondria-enriched fractions were obtained from control (CC) and three different ND1-null (OS) cybrids in absence (untreated, UT) of 50 µg/mL chloramphenicol and at different times (0, 6, 16 and 24 h) after chloramphenicol removal. Fully assembled CI and sub-complexes (Sb1-2-3) are indicated by dotted lines. One representative experiment of three is shown. (**D**) 2D BN/SDS-PAGE, followed by western blotting using an antibody against SDHA, carried out in mitochondria-enriched fractions isolated from CC and OS clone in the absence (untreated, UT) of 50 µg/mL chloramphenicol and at different times (0, 6, 16 and 24 h) after chloramphenicol removal. Fully assembled CII is indicated by dotted lines. One representative experiment of three is shown. (**E**) Mean incorporation rates of NDUFS3 in fully assembled CI after chloramphenicol removal. The signal was quantified on the level of SDHA, a subunit of CII. Mean values are expressed as percentages relative to untreated cells (UT, 100%).

To evaluate the role and the amount of the human ND1 protein required for a proper CI biogenesis, a series of osteosarcoma-derived cybrid clones bearing different loads of the frameshift m.3571insC/*MT-ND1* mutation were generated (Table 1). Homoplasmic cell lines (OS) clearly showed that this mutation induced the complete lack of ND1 subunit, as previously reported [18] (Figure 1A). CI in-gel activity (CI-IGA) and western blot analysis against NDUFA9 (NADH dehydrogenase:ubiquinone 1 alpha subcomplex subunit 9) revealed the absence of a fully assembled and functional CI in clones lacking ND1 (Figure 1A). This finding was confirmed by using an antibody against the NDUFS3 (NADH dehydrogenase:ubiquinone iron-sulfur protein 3) subunit, which selectively traces sub-complexes of the Q-module (Sb1-2-3; Figure 1B) [19]. In OS clones, 2D BN/SDS-PAGE analysis showed accumulation of Sb1-2-3 in parallel with the lack of fully assembled CI, a pattern similar to that observed in mtDNA-deprived Rho 0 cells (Figure 1B).

**Figure 2:**
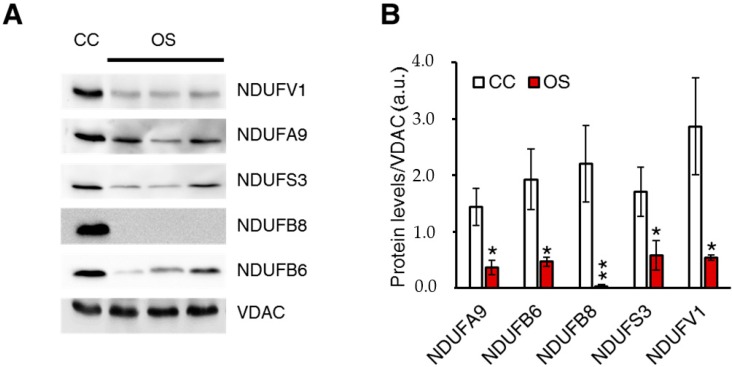
ND1 ablation impacts on steady state levels of nDNA-encoded complex I (CI) subunits. (**A**) Immunoblotting of CI nuclear-encoded subunits in crude mitochondria from control (CC) and three different ND1-null (OS) cybrids. VDAC1 was used as a loading control. One representative experiment of three is shown. (**B**) Densitometric quantification of western blot analysis of CI nuclear-encoded subunits. Data are mean ± SEM (*n* = 3, * *p*-value < 0.05; ** *p*-value < 0.001).

To assess whether CI assembly kinetics was modified upon ablation of ND1, controls (CC) and OS clones were treated with chloramphenicol, a reversible inhibitor of mitochondrial translation inducing the depletion of mtDNA-encoded proteins, and CI re-assembly was followed at different time points by 2D BN/SDS-PAGE (Figure 1C). After 4 days of treatment with chloramphenicol, no band corresponding to the fully assembled CI was detectable either in mutant or in controls clones (T = 0 h in Figure 1C). However, in agreement with previous reports [19], residual NDUFS3 spots were detected in Sb1-3 (Figure 1C), indicating that CI assembly is unable to proceed beyond its early stages when the synthesis of mtDNA-encoded subunits is inhibited. At 16 and 24 h after chloramphenicol withdrawal, the spot corresponding to fully assembled CI was observed only in CC (Figure 1C–E), indicating that the biogenesis of the holoenzyme was strongly impaired in absence of ND1. Nonetheless, the recovery of fully assembled CI in CC clones after 24 h of chloramphenicol withdrawal was still not complete, since the amount of CI at this time point was about 50% of its steady state levels. As expected, chloramphenicol treatment did not affect the levels of entirely nuclear-encoded CII (Figure 1D). Then, to investigate the effect of m.3571insC/*MT-ND1* mutation on steady-state levels of several nuclear-encoded CI subunits and on CI assembly/function, crude-mitochondria fractions were solubilized. Western blot analysis using the antibodies against subunits belonging to the three different CI modules NDUFV1, NDUFA9, NDUFS3, NDUFB8, and NDUFB6 showed a significant reduction of these subunits in OS compared to CC clones (Figure 2A,B). Taken together, these data suggest that human ND1 subunit is essential to drive a proper CI biogenesis and that its absence affects the stability of nDNA-encoded subunits belonging to different CI functional modules.

### 2.2. Small Amounts of Wild Type ND1 Subunit Are Sufficient to Partially Recover CI Disruption

**Figure 3:**
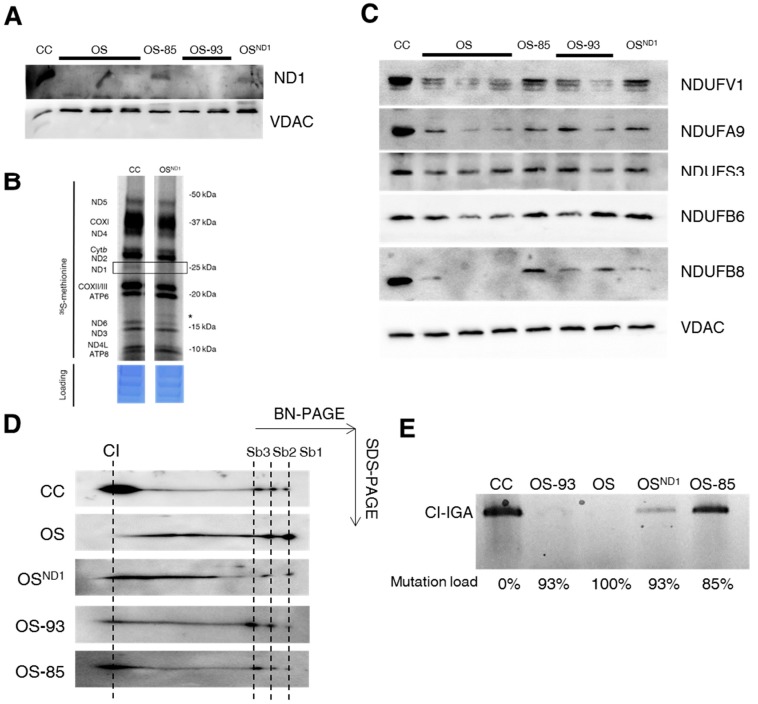

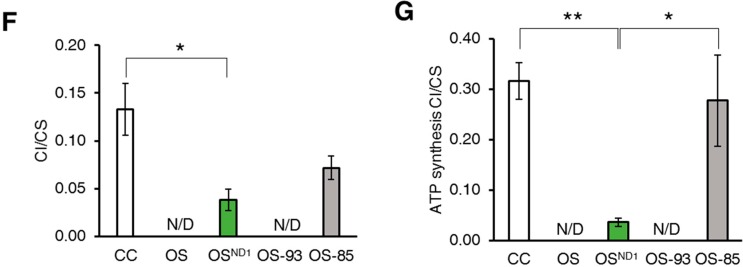
Low levels of wild type ND1 partially rescue CI assembly and function. (**A**) Western blot analysis of ND1 levels in crude mitochondria from control (CC), ND1-null (OS), 93% ND1 mutant (OS-93), 85% ND1 mutant (OS-85) and allotopically complemented (OS^ND1^) cybrids. VDAC1 was used as loading control. One representative experiment of three is shown. (**B**) Mitochondrial in vitro translation. The autoradiogram displaying the radiolabeled mitochondrial translation products and the corresponding Coomassie blue-stained gel as a control for loading are shown. The boxed section correspond to the endogenous ND1 subunit. A small protein product of about 15 kDa, indicated by an asterisk, is detectable in OS^ND1^. This band may be the *bona fide* truncated ND1 subunit. (**C**) Western blot analysis of steady state levels of CI nuclear-encoded subunits in crude mitochondria from control (CC), ND1-null (OS), 93% ND1 mutant (OS-93), 85% ND1 mutant (OS-85), and allotopically complemented (OS^ND1^) cybrids. VDAC1 was used as a loading control. One representative experiment of three is shown. (**D**) 2D BN/SDS-PAGE followed by western blotting using an antibody against NDUFS3 subunit performed on mitochondria-enriched fractions. Fully assembled CI and sub-complexes (Sb1-2-3) are indicated by dotted lines. One representative experiment of three is shown. (**E**) CI-IGA assay after separation of mitochondria-enriched fractions by hrCNE. One representative experiment of three is shown. (**F**) CI redox specific activity (rotenone-sensitive) normalized on citrate synthase (CS) activity. Data are mean ± SD (*n* = 5, * *p*-value < 0.05). (**G**) Mitochondrial ATP synthesis rate driven by CI substrates. Data are normalized on CS activity and are mean ± SD (*n* = 5, * *p*-value < 0.05; ** *p*-value < 0.001).

**Figure 4:**
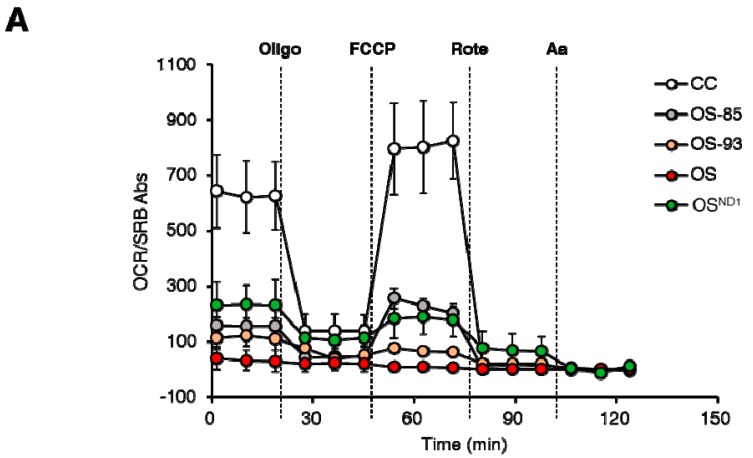

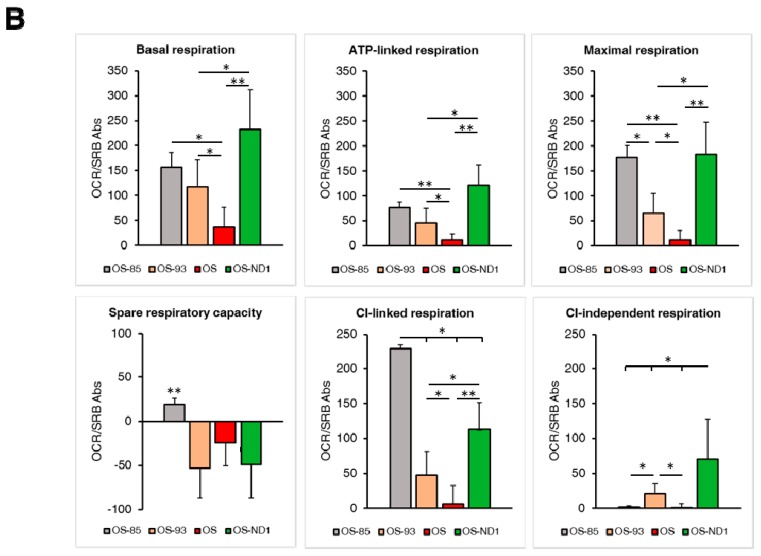
Mitochondrial respiration depends on ND1 abundance. (**A**) Oxygen consumption rate (OCR) profiles performed in 10 mM glucose medium upon injection of 1 µM oligomycin (Oligo), variable concentration of carbonyl cyanide 4-(trifluoromethoxy)phenylhydrazone (FCCP) determined by titration, 1 µM rotenone (Rote), and 1 µM antimycin A (Aa). Data (mean ± SD, *n* ≥ 4) are expressed as OCR (picomoles of O_2_ per minute) normalized on SRB absorbance. All the data points from CC are statistically significant compared to all the other cell lines (**B**) Basal, ATP-linked and maximal respiration, spare respiratory capacity, rotenone sensitive, and insensitive OCR of mutant cells with different amount of ND1. Data (mean ± SD, *n* ≥ 4, * *p*-value < 0.05; ** *p*-value < 0.001) are expressed as OCR (picomoles of O_2_ per minute) normalized on SRB absorbance.

In order to provide a proof of concept to our findings, we exploited cells in which ND1 was allotopically complemented with a nuclear-encoded version of *MT-ND1* gene (OS^ND1^) [21], which allowed us to recover wild type ND1 through expression of a cytosolically synthesized protein. We expressed allotopic ND1 in OS-93 cells carrying the m.3571insC/*MT-ND1* mutation at 93% of heteroplasmy (Table 1). Similar to OS clones, OS-93 clones did not express ND1 (Figure 3A). Moreover, we also used OS-85 clones with 85% of heteroplasmic mutation as a control, since they naturally re-express ND1 protein (Table 1, Figure 3A). Allotopic expression of ND1 recovered the levels of this subunit in OS^ND1^ despite the lack of the endogenous mtDNA-encoded protein (Figure 3A,B, boxed section). The amount of allotopically expressed ND1 was very low compared to controls but sufficient to increase the levels of some nDNA-encoded CI subunits, such as NDUFV1, NDUFA9, and NDUFB6 (Figure 3C) and to permit a proper assembly of CI (Figure 3D), partially overcoming the stall during its biogenesis (Figure 1C). Indeed, OS^ND1^ clones showed decreased amount of low molecular weight subassemblies Sb1-3 compared to both OS and OS-93 clones, while high molecular weight intermediates and fully assembled CI increased (Figure 3D). Allotopic expression of ND1 was also able to partially recover CI function in terms of NADH dehydrogenase activity (Figure 3E,F) and to restore part of the membrane electrochemical proton gradient to sustain the ATP synthase activity (Figure 3G). On the other hand, OS-85 clones displayed an increase of all nDNA encoded CI subunits compared to OS-93 and OS clones (Figure 3C) and 2D BN/SDS-PAGE experiments showed a profile comparable to controls with very low amount of Sb1-3 and an almost complete recovery of fully assembled CI (Figure 3D), indicating that 15% of wild type mtDNA molecules are sufficient to restore the assembly of CI. Moreover, CI-IGA, rotenone-sensitive CI activity and CI-driven ATP synthesis were undetectable in OS and OS-93 (Figure 3E–G), whereas OS-85 showed a recovery of about 50% in rotenone sensitive NADH:DB oxidoreductase activity and CI-IGA (Figure 3E,F) and resulted comparable to controls in terms of CI-driven ATP-synthesis (Figure 3G). To further define the biochemical effect of different mutation load, we performed a microrespirometry assay on intact cells using the Seahorse platform. Control cell line showed a significantly higher respiration rate (basal, ATP-linked, and maximal respiration) compared to the mutant cell lines, namely OS-85, OS-93 and OS (Figure 4A). Such respiration derives completely from CI activity, as the rotenone-insensitive oxygen consumption rate (OCR) was almost undetectable (Figure 4A). On the other hand, OS clones do not respire and not respond to the injection of OXPHOS inhibitors and mitochondrial uncoupling agent (Figure 4A,B). OS-85 and OS-93 showed similar basal and ATP linked respiration (Figure 4B). However, OS-93 do not display any spare respiratory capacity, as its OCR cannot be further stimulated by chemical uncoupling (Figure 4B). The allotopic expression of ND1 allowed a partial recovery of OCR to levels similar to OS-85 (Figure 4B). CI-linked respiration was gradually increasing in inverse correlation with the mutation load and OS^ND1^ clone stands between OS-93 and OS-85. Interestingly, while OS-85 respiration is completely rotenone-sensitive, OS-93 and OS^ND1^ also showed some rotenone-insensitive OCR, suggesting the activation of alternative pathways to feed CIII with reduced ubiquinone (Figure 4B). This phenotype warrants further investigations.

Overall, these results suggest that the m.3571insC genetic threshold for collapse of CI in terms of structure and function ranges between 93% and 85%. Hence, a low amount of ND1 is sufficient to drive CI formation towards high molecular weight assembly intermediates, leading to a functional holoenzyme.

### 2.3. Lack of ND1 Hampers Supercomplexes Formation and Stability

**Figure 5:**
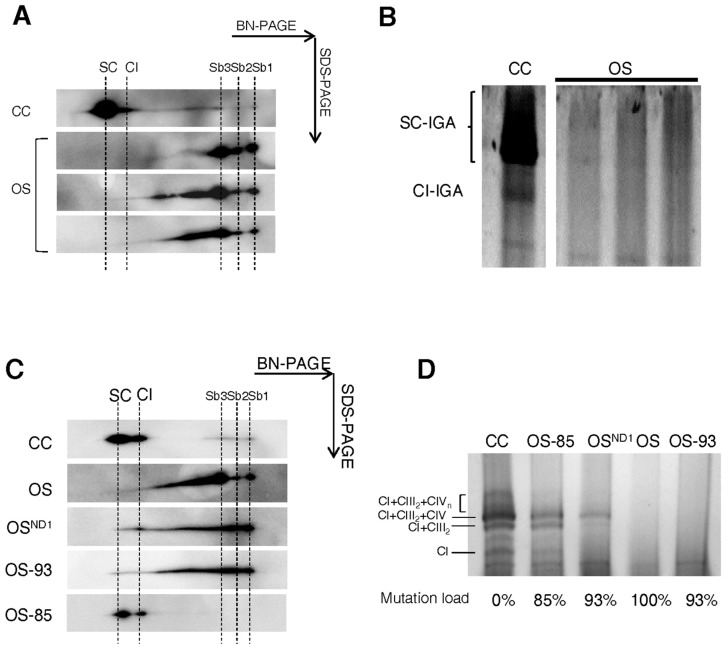
ND1 is required for respirasome CI+CIII_2_+CIV formation and stability. (**A**) 2D BN/SDS-PAGE and western blotting of respirasome (indicated as SC) performed on mitochondria-enriched fractions using an antibody against NDUFS3. Fully assembled CI is indicated as CI; sub-complexes of CI are indicated as Sb1-2-3. One representative experiment of three is shown. (**B**) CI-IGA of supercomplexes (SC) and isolated CI performed on mitochondria-enriched fractions. One representative experiment of three is shown. (**C**) 2D BN/SDS-PAGE and western blotting for supercomplexes (SC) performed on mitochondria-enriched fractions using an antibody against NDUFS3. CI sub-complexes are indicated as Sb1-2-3. One representative experiment of three is shown. (**D**) CI-IGA of supercomplexes (indicated as CI+III_2_+IV_n_, CI+III_2_+IV or CI+CIII_2_) and isolated CI (CI) of mitochondria-enriched fractions. One representative experiment of three is shown.

As previously detailed, CI may act as a scaffold for the incorporation of CIII_2_ and several units of CIV in high molecular weight supercomplexes [10]. In particular, CI and CIII_2_ association is preferentially maintained even when a partial loss of CIII occurs [22]. Thus, to understand the minimum amount of ND1 required for supercomplexes biogenesis and stability, we analyzed their assembly pattern in mitochondria-enriched fractions from our clones. Upon 2D BN/SDS-PAGE separation and immunodetection of NDUFS3, accumulation of Sb1-2-3 and lack of fully assembled CI were found in OS compared to CC (Figure 5A). Supercomplexes I+III_2_+IV_n_ and their CI-IGA were detected in CC only (Figure 5A,B). Similarly, no functional supercomplexes were detected in OS-93 cells (Figure 5C,D), indicating that 7% of wild type mtDNA molecules are not sufficient to reassembly supercomplexes with NADH dehydrogenase activity. Although the allotopic expression of ND1 in OS^ND1^ cells was able to recover only low levels of assembled supercomplexes I+III_2_+IV_n_ (Figure 5C), a band with NADH dehydrogenase activity was present (Figure 5D). On the other hand, OS-85 clones showed only fully assembled supercomplexes and no accumulation of subcomplexes containing NDUFS3 subunit (Figure 5C). Interestingly, CI-IGA varied in correlation with the mutation load and the amount of ND1 subunit (Figure 5D). In control cell lines multiple bands corresponding to isolated CI and supercomplexes with different CIII and CIV stoichiometry were detectable, while OS and OS-93 clones completely lack of CI-IGA (Figure 5D). OS-85 showed NADH dehydrogenase activity in the bands corresponding to supercomplex I+III_2_+IV (the respirasome) and I+III_2_. These cells also showed very faint bands corresponding to isolated CI and to supercomplexes with higher molecular weights (Figure 5D). On the other hand, the low allotopic expression of ND1 lead to the formation of respirasome only and no additional bands positive for NADH dehydrogenase activity were detected (Figure 5D). These data confirm that the mutation threshold resides between 93–85% of wild type *MT-ND1*, since 15% of wild type mtDNA is sufficient to restore the correct pattern of supercomplexes, but also indicate that respirasome is the preferential or most stable form of supercomplexes, as it is the first supercomplex reassembled when CI is a limiting component. Overall, these data support the hypothesis of CI as a scaffold for supramolecular organization of respiratory complexes [10].

## 3. Discussion

In this study, we exploited a set of cell models to investigate how the frameshift m.3571insC/*MT-ND1* mutation modulated the abundance of human ND1 subunit and, thus, impacts on the biogenesis and stability of isolated CI and its combination with CIII and CIV in supramolecular entities. So far, this mutation has never been reported as germ line, while it has been found in multiple cases of oncocytic tumors belonging to different tissues [18,23,24,25] and one case of glioma [26], clearly indicating the detrimental nature of its functional effects on respiratory CI and highlighting its *oncojanus* role in the context of tumor progression. We show that the homoplasmic frameshift m.3571insC/*MT-ND1* mutation, which cause the lack of human ND1, severely affects CI assembly, leading to the accumulation of non-functional sub-complexes and hampering the formation of a fully organized functional respirasome. Complex I biogenesis is an intricate modular process requiring the coordinated expression of structural subunits and assembly factors, and in which subcomplexes are preassembled and then combined together to form the mature complex [3]. Through the years, different assembly models have been proposed. The early steps of CI biogenesis involve the preassembly of the Q-module containing NDUFS3 and other nuclear-encoded subunits [19]. Subsequently, ND1 is incorporated in this intermediate and drives the formation of the proximal part of the membrane arm in contact with the Q-module [3]. Previous studies demonstrated that the lack of ND1 in bacteria results in severe alteration of CI assembly [27,28]. Similar results have been recently reported in human cells, where the absence of ND1 has been shown to affect the early stages of CI biogenesis, leading to the disruption of the fully assembled complex [29]. Accordingly, here we demonstrate that when ND1 is ablated the biogenesis of CI is stalled at its early stages, as clearly indicated by the accumulation of low molecular weight, Sb1-3 assembly intermediates containing NDUFS3, corresponding to the 99, 129 and 170 kDa intermediates of the most updated model of CI assembly [3]. Such a pattern is indistinguishable from the one displayed by mtDNA-devoid cells [30]. The 2D BN/SDS-PAGE analysis of ND1 defective cells occasionally showed the occurrence of assembly intermediates with higher molecular weight containing NDUFS3 comparable with subcomplexes 4 and 5 previously identified by Vogel and colleagues [19] and detected also in Rho 0 cells [30]. These data may underlie the possible presence of instable high molecular subcomplexes even when ND1 is missing, and warrant further investigations. Similarly to Lim and colleagues [29], we observed that the steady state levels of several nuclear subunits NDUFS3, NDUFA9, NDUFB8, and NDUFV1 were reduced in cells lacking ND1, suggesting that all the functional modules of CI are altered when its assembly is stalled at early stages. It has been proposed that the absence of ND1 does not impact on the abundance of NDUFB6 subunit because of its incorporation into a subcomplex of 460 kDa, which is found accumulated in cells missing ND1 [29]. However, in our cell models, we observed reduced levels of NDUFB6 when ND1 is ablated, suggesting that the formation of a 460 kDa assembly intermediate may be perturbed or that steady state levels of NDUFB6 do not reflect the accumulation of such intermediate. It is important to note that the assembly step in which incorporation of NDUFB6 occurs during CI biogenesis is still controversial. According to the most updated CI assembly model, the integral membrane subunit NDUFB6 is incorporated in assembly intermediates of 230 and 680 kDa containing part of the proximal membrane arm [3], which may correspond to the assembly intermediates containing NDUFB6 during the reassembly of CI after chloramphenicol treatment [31]. However, NDUFB6 has been also found in an assembly intermediate of 460 kDa containing, among others, ND2, ND3, ND4L, and ND6 subunits [4,29], suggesting a possible alternative mechanism for its insertion during CI biogenesis. Interestingly, we have previously shown that CI-defective tumors carrying disruptive mtDNA mutations, also affecting *MT-ND1*, mostly showed a negative staining for NDUFB6 in vivo [23,32], supporting the idea that such mutations impact on the levels of this subunit.

In this study, we reported for the first time that low amounts of wild type ND1 are sufficient to partially overcome the CI biogenesis stall, allowing its maturation and slightly ameliorating the defective bioenergetic phenotype. A similar rescue is also apparent when the m.3571insC mutation load is around 85%; whereas when mutant mtDNA molecules are around 93%, CI NADH dehydrogenase activity and CI-driven ATP synthesis cannot be detected, despite the presence of a faint band corresponding to the fully assembled enzyme. Based on these data, we set a threshold for the m.3571insC mutation between 93% and 85%, above which CI is not properly assembled leading to a severe energetic dysfunction. It is well known that mtDNA mutations must surpass a certain threshold to exert their functional effects and that the phenotypic threshold depends on the mutation type and on the affected tissue [33,34,35,36,37] but usually ranges between 70% and 90% mutant molecules. In the case of the m.3571insC/*MT-ND1* mutation, genetic, enzymatic, and phenotypic thresholds seem to parallel. Indeed, we have previously demonstrated that when the m.3571insC mutation surpasses the threshold of 85–93%, mutant molecules cells displayed mitochondrial structural derangement and energetic crisis, leading to the activation of the cell energy sensor AMP-activated kinase (AMPK) [24,38]. Cells surpassing the mutation threshold showed also a profound metabolic remodeling and chronic destabilization of the Hypoxia Inducible Factor 1α (HIF-1α), preventing the hypoxic and metabolic adaptation necessary for cancer cells to proceed toward malignancy [23,24,38]. In this context, it is of pivotal importance to precisely define the genetic and functional threshold, since very small differences in terms of mutant load may cause divergent clinical phenotypes.

Lastly, this study also demonstrates that such small differences in ND1 abundance impacts on respirasome formation and stability. Two models of supercomplexes assembly have been proposed which differ for the requirement of a complete CI to form supramolecular structures [10,39]. In particular, a 830 kDa CI sub-assembly intermediate lacking the NADH dehydrogenase module has been proposed to be required for the proper biogenesis of the respirasome, acting as a scaffold for the incorporation of free CIII_2_ and CIV [10]. Since the presence of the m.3571insC/*MT-ND1* mutation above threshold disrupt the CI assembly at its early stages, our data do help to discriminate between the two models. In agreement with our work, it has been recently reported that the homoplasmic m.3571insC/*MT-ND1* mutation induced the disruption of supercomplexes CI+CIII_2_+CIV and CI+CIII_2_ [29]. Our work not only confirms these results, but also provides the evidence that a small amount of ND1 (in the range between 85–93% of mutation load) is able to recover the supercomplexes assembly and, most importantly, function. Moreover, our results highlight the preferential aggregation of respiratory complexes in supramolecular structures. In fact, when wild type ND1 is expressed at low levels, supercomplexes were found almost exclusively in the form CI+III_2_+IV, and isolated CI is not detected. Beside the respirasome assembly, increasing levels of ND1 determined also the generation of CI+III_2_ supercomplex and very low levels of isolated CI. Hence, our data strongly support the idea that respirasome has a functional significance, although this point remains to be elucidated [14].

## 4. Methods

### 4.1. Cell Lines and Culture Conditions

Cell lines used for this study are 143B TK^−^ osteosarcoma (OS)-derived cybrids carrying the m.3571insC frameshift mutation in *MT-ND1* with different mutant loads (Table 1). This mutation inserts a premature stop codon at amino acid position 101. We selected three homoplasmic clones (OS) and two heteroplasmic clones bearing 85% and 93% of mutated *MT-ND1* (OS-85 and OS-93, respectively [23]). All these clones were selected by incubating OS cells with ethidium bromide (50 ng/mL) for 10 days, in order to reduce the mtDNA copy number, and then allowed to repopulate. OS-93 clones allotopically complemented with wild type cytosolically translated ND1 (OS^ND1^) and controls (CC) were previously described [21]. 143B TK^−^ depleted of mtDNA (Rho 0 cells) were used as CI-negative controls. Cells were grown in Dulbecco’s modified Eagle medium (DMEM) supplemented with 10% fetal calf serum, 2 mM l-glutamine, 100 U/mL penicillin, 100 µg/mL streptomycin, 50 µg/mL uridine (Life Technologies, Milan, Italy), in an incubator with a humidified atmosphere of 5% CO_2_ at 37 °C.

### 4.2. Mitochondrial DNA Sequencing and Low Heteroplasmy Detection

The mutation load of m.3571insC/*MT-ND1* was assessed using Fluorescent PCR (F-PCR) and Denaturing High Performance Liquid Chromatography (DHPLC) as previously reported [20]. Each analysis was performed in triplicate. Primers and conditions are available on request.

### 4.3. Mitochondrial-Enriched Fraction and Crude Mitochondria Preparation

Mitochondria-enriched fractions were obtained by subcellular fractionation (5–10 × 10^6^ cells) in presence of digitonin (50 μg/mL) [40]. Crude mitochondria were obtained from 15–20 × 10^6^ cells. The cell pellet was suspended in ice-cold buffer containing 200 mM mannitol, 70 mM sucrose, 1 mM EGTA, 10 mM HEPES (pH 7.6), and mechanically disrupted with a glass/teflon Potter-Elvehjem homogenizer. Differential centrifugations (600× *g* for 10 min at 4 °C followed by 14,000× *g* for 10 min at 4 °C) were performed to separate crude mitochondria from other sub-cellular fractions. Samples were stored at −80 °C.

### 4.4. SDS-PAGE and Western Blot Analysis

Mitochondrial proteins were extracted from crude mitochondria using 2% *N*-dodecyl β-d-maltoside (DDM). Mitochondrial extracts were separated by 10% SDS-PAGE and transferred onto a nitrocellulose membrane. Membranes were incubated overnight at 4 °C with antibodies against subunits belonging to different functional modules of CI in order to understand which module is affected by the presence of the m.3571insC/*MT-ND1* mutation: NDUFA9 (Q-module, 1:1000, Abcam, Cambridge, UK), NDUFS3 (Q-module, 1:1000, Abcam, Cambridge, UK), NDUFV1 (N-module, 1:500, Sigma Aldrich, Milan, Italy), NDUFB8 (P-module, 1:1000, Life Technologies, Milan, Italy), NDUFB6 (P-module, 1:1000, Life Technologies, Milan, Italy), and ND1 (P-module, 1:1000, homemade by A. Lombes). VDAC1 (1:1000, Abcam, Cambridge, UK) was used as loading control. Primary antibodies were visualized using horseradish peroxidase-conjugated secondary antibodies (1:2000, Jackson ImmunoResearch, West Grove, PA, USA). Chemiluminescence signals were acquired with Gel Logic 1500 molecular imaging apparatus (Kodak, Rochester, NY, USA). Densitometric quantifications were carried out by using Image J 1.48 v [41].

### 4.5. High-Resolution Clear Native PAGE (hrCNE)

For CI-IGA assay and western blotting analysis, mitochondrial enriched fractions were suspended in mitochondrial buffer (750 mM aminocaproic acid, 50 mM Bis-Tris, pH = 7) and solubilized by adding DDM/protein ratio of 2.5 (g/g). Suspension was incubated on ice for 10 min and then centrifuged at 13,000× *g* for 15 min. Aliquots of supernatants (80 µg protein) were separated by 4–16% first dimension hrCNE gradient gel and CI-IGA was detected or transferred onto a nitrocellulose membrane, as previously described [38].

### 4.6. First Dimension Blue Native-PAGE (1D BN-PAGE)

Native complexes and supercomplexes were analyzed as previously described [40]. Briefly, for isolated complexes, mitochondrial enriched fractions were suspended in mitochondrial buffer (750 mM aminocaproic acid, 50 mM Bis-Tris, pH = 7) and solubilized by adding DDM/protein ratio of 2.5 (g/g). Suspension was incubated on ice for 10 min and then centrifuged at 13,000× *g* for 15 min. Aliquots of supernatants (100 µg protein) were separated by 4–15% first dimension Blue Native gradient gel. Supercomplexes analysis was performed as previously described with minor modifications [40]. Briefly, mitochondria-enriched fractions were suspended in 340 mM K-acetate, 70 mM HEPES (pH 7.4) plus 25% glycerol, 2.3 mM PMSF, 47 mg/mL digitonin and incubated at 4 °C for 30 min. Samples were centrifuged for 2 min at 600× *g* at 4 °C and supernatants (100 µg protein) were separated by 3–12% BN-PAGE as described above for Blue Native-PAGE. After electrophoresis, gels were processed for CI-IGA, western blotting or second dimension SDS-PAGE (2D BN/SDS-PAGE).

### 4.7. 2D BN/SDS-PAGE

Lanes excised from 1D BN-PAGE gradient 4–12% or 3–12% were treated with denaturing buffer containing 1% sodium dodecyl sulfate (SDS) and 0.1% β-mercaptoethanol for 90 min and then separated on 10% SDS-PAGE as previously described [40]. After electrophoresis, gels were processed in western blotting as described above.

### 4.8. Complexes Re-Assembly Kinetics Assay

To follow the assembly kinetics of CI and CII, cells were incubated for 4 days with culture medium containing 50 µg/mL chloramphenicol, a reversible inhibitor of mitochondrial translation [42]. Cells were then cultured in normal medium and harvested at indicated time points. Mitochondria-enriched fractions were isolated as described above and analyzed by 2D BN/SDS-PAGE followed by western blot analysis with an antibody against NDUFS3 (1:1000, Abcam, Cambridge, UK) or SDHA (1:10,000, Life Technologies, Milan, Italy).

### 4.9. In Vitro Mitochondrial Translation Assay

Translation of mtDNA encoded proteins was assayed by ^35^S-methionine labelling. Cells (80% confluent) were washed twice and incubated with a methionine/cysteine-free DMEM for 15 min at 37 °C at 5% CO_2_. Incubation medium was removed and replaced with 1 mL of methionine-free DMEM supplemented with 5% dialyzed serum, 1 mM sodium pyruvate, 2 mM l-glutamine, 96 µg/mL cysteine, and 100 µg/mL emetine dihydrochloride. Cells were incubated for 20 min at 37 at 5% CO_2_ before addition of 166 µCi/mL [^35^S]-methionine to the culture medium. Labelling was performed for 60 min at 37 °C at 5% CO_2_, then cells were harvested, and washed with ice-cold phosphate buffered saline (PBS). Cell pellet was suspended in PBS containing 0.1% DDM and 2 U/µL benzonase and incubated on ice for 30 min. Then, 1% SDS was added to the samples and protein quantification was performed using the DC protein assay (Biorad, Hercules, CA, USA). Protein samples (30 µg) were separated on 10–20% precasted SDS-PAGE gels (Life Technologies, Milan, Italy). Gels were stained with Coomassie Brilliant Blue for 10 min, destained for 1 h and scanned. Gels were dried before exposure to a Phosphorimager screen. Radiolabelled proteins were analyzed by using a Typhoon™ Phosphorimager system and an ImageQuant software (Molecular Dynamics, GE Healthcare, Milan, Italy).

### 4.10. Spectrophotometric Assays of Respiratory Complexes Activity

Crude mitochondria were suspended in 10 mM Tris (pH 7.6) and kept at −80 °C. Redox enzymatic activities were measured at 37 °C using a dual-wavelength spectrophotometer (V550 Jasco Europe, Cremella, Lecco, Italy). CI activity (NADH:DB:DCIP oxidoreductase) was assessed in 25 mM potassium phosphate buffer (pH = 7.8), containing 60 µM 2,6-dichlorophenolindophenol (DCIP) (λ = 600 nm; ɛ_DCIP_: 19.1 mM^−1^·cm^−1^), 70 µM decylubiquinone (DB), 3.5 g/L BSA, and 200 µM NADH, as previously described [40]. CI activity was measured in absence and in presence of 1 µM rotenone. Data were normalized to protein content and citrate synthase (CS) activity, determined as previously reported [40].

### 4.11. Mitochondrial ATP Synthesis

The rate of mitochondrial ATP synthesis was measured in digitonin-permeabilized cells as previously described [40]. Briefly, after trypsinization, cells (10 × 10^6^/mL) were suspended in a buffer containing 150 mM KCl, 25 mM Tris-HCl, 2 mM EDTA (ethylenediaminetetraacetic acid), 0.1% bovine serum albumin, 10 mM potassium phosphate, 0.1 mM MgCl_2_, pH 7.4, kept at room temperature for 15 min, then incubated with 50 μg/mL digitonin until 90–100% of cells were positive to Trypan Blue staining. Aliquots of 3 × 10^5^ permeabilized cells were incubated in the same buffer in the presence of the adenylate kinase inhibitor P^1^,P^5^-di(adenosine-5′) pentaphosphate (0.1 mM) and CI substrates 1 mM malate plus 1 mM pyruvate. After the addition of 0.2 mM ADP, chemiluminescence was measured during time (Sirius L Tube luminometer, Titertek-Berthold, Pforzheim, Germany). The chemiluminescence signal was calibrated with an internal ATP standard after the addition of 10 μM oligomycin. The rates of the ATP synthesis were normalized to protein content and CS activity.

### 4.12. Microrespirometry

Oxygen consumption rate (OCR) in adherent cells was measured with an XFe24 Extracellular Flux Analyzer (Seahorse Bioscience, Billerica, MA, USA), as previously described [38]. Briefly, cells were seeded in XFe24 cell culture microplates at 3 × 10^4^ cells/well in 200 μL of complete medium and incubated at 37 °C in 5% CO_2_ for 24 h. Assays were initiated by replacing the growth medium in each well with 570 μL of unbuffered DMEM supplemented with 10 mM glucose and 5 mM Na-pyruvate pre-warmed at 37 °C. Cells were incubated at 37 °C for 30 min to allow temperature and pH equilibration. After an OCR baseline measurement, 1 μM oligomycin, variable concentration of FCCP, and 1 μM rotenone and antimycin A were sequentially injected. FCCP optimal concentration was previously determined for each cell line by titration following the manufacturer’s protocol (0.5 μM for CC, 0.2 μM for OS-85 and OS^ND1^, 0.1 μM for OS-93 and OS). At the end of each experiment, the medium was removed and protein content was determined by SRB assay. OCR data (pmol/min) are normalized on SRB absorbance as previously described [38].

### 4.13. Statistical Analyses

One way ANOVA or Student’s *t*-test were applied for statistical analyses. Data were considered statistically significant for *p*-values ≤ 0.05.

## 5. Conclusions

Overall, our data prove that the amount of ND1 subunit is deeply involved in the structural remodeling of respiratory chain and, hence, mitochondrial energetic function, conferring to this protein a prominent role in the pathology of mitochondria-related diseases.

## Figures and Tables

**Table 1:** Mutation loads for m.3571insC/*MT-ND1* mutation in cell models used in this study determined by F-PCR [20].

Cell Line	Mutation Load
CC	0%
OS	100%
OS-93	92.8 ± 0.3%
OS-85	85.3 ± 0.4%
OS^ND1^	93.4 ± 1.3%
Rho 0	no mtDNA

## Abbreviations

1D BN-PAGE	1 dimension Blue Native PolyAcrylamide Gel Electrophoresis
2D SDS/BN-PAGE	2 dimension SDS/Blue Native PolyAcrylamide Gel Electrophoresis
ADP	Adenosine diphosphate
AMP	Adenosine monophosphate
AMPK	AMP-activated kinase
ATP	Adenosine triphosphate
BSA	Bovine Serum Albumine
CI	Complex I
CII	Complex II
CI-IGA	Complex I In-gel Activity
CIII	Complex III
CIV	Complex IV
CS	Citrate Synthase
CV	Complex V
DB	Decylubiquinone
DCIP	2,6-dichlorophenolindophenol
DDM	n-dodecyl β-maltoside
DHPLC	Denaturing High Performance Liquid Chromatography
DMEM	Dulbecco’s Modified Eagle Medium
EDTA	Ethylenediaminetetraacetic acid
EGTA	Ethylene glycol-bis (β-aminoethyl ether)-*N*,*N*,*N*′,*N*′-tetraacetic acid
F-PCR	Fluorescent PCR
HEPES	4-(2-hydroxyethyl)-1-piperazineethanesulfonic acid
HIF-1α	Hypoxia Inducible factor 1α
hrCNE	High resolution Clear Native Electrophoresis
mtDNA	Mitochondrial DNA
NADH	Reduced nicotinamide adenine dinucleotide
nDNA	Nuclear DNA
OS	Osteosarcoma
OXPHOS	Oxidative phosphorylation
PBS	Phosphate buffered saline
Sb	Subcomplex
SDS-PAGE	Sodium dodecyl sulfate PolyAcrylamide Gel Electrophoresis
